# Benchmarking Classical and Quantum Machine Learning for Potency Prediction across Ten Therapeutically Relevant Targets

**DOI:** 10.1021/acsmedchemlett.5c00724

**Published:** 2026-07-07

**Authors:** Shiv Garg, Sara Garg

**Affiliations:** † 1372 Georgia Institute of Technology, Atlanta, Georgia 30332, United States; ‡ 310202 Emory University, Atlanta, Georgia 30322, United States

**Keywords:** Computational Chemistry, Cheminformatics, Drug Discovery, Virtual Screening, Machine Learning, Quantum Computing

## Abstract

Quantum machine learning (QML) is widely proposed as a successor to classical models in cheminformatics, but rigorous benchmarks under realistic contemporary data constraints remain scarce. We compared two quantum classifiers (quantum support vector machine, variational quantum classifier) against gradient boosting, random forest, and neural network baselines across ten therapeutically diverse protein targets (45–18,286 compounds), using Bemis–Murcko scaffold-stratified cross-validation. Across 48 valid target-threshold tasks, no model class globally dominated (best-quantum versus best-classical Wilcoxon p = 0.189). Classical ensembles excelled on data-rich, structurally coherent targets (e.g., BACE1), while quantum kernels held a marginal edge on sparse, structurally bimodal targets (e.g., Mtb DprE1). A potential predictor of quantum advantage was the per-target best-quantum/best-classical model Cohen’s κ gap (Spearman ρ = +0.685, p = 0.029), suggesting that decision calibration, not ranking, drives QML’s edge. We propose the κ gap as a preliminary low-cost screening criterion for prioritizing QML in early stage discovery campaigns.

## Graphical abstract

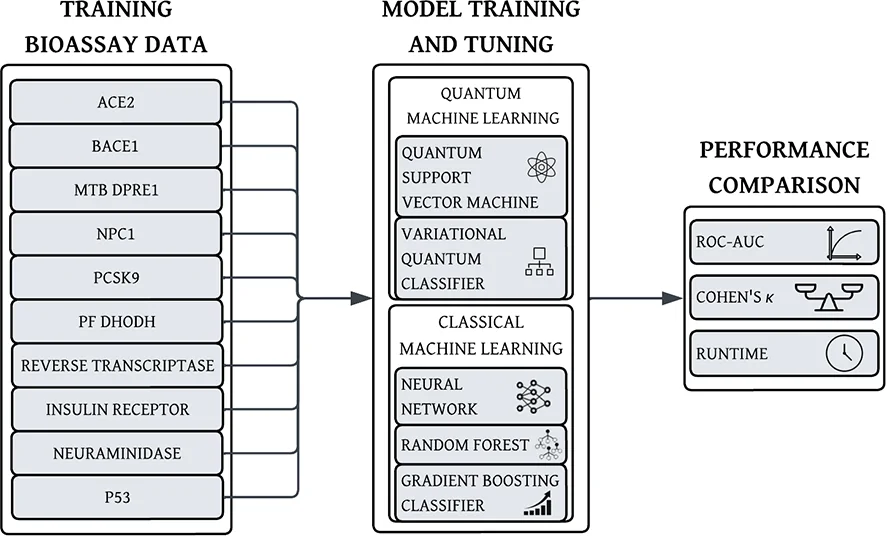

While drug discovery is increasingly foundational to global public health, it remains a long, expensive, and high-attrition enterprise: only 12% of proposed drugs reach market approval, and bringing a single new drug to market takes 10–15 years and approximately 2.5 billion dollars to develop.^1^ Computational prioritization of compounds for synthesis is one of the few levers that can meaningfully shift this economics by reducing the number of molecules that need to be made and tested experimentally.

Quantitative structure–activity relationship (QSAR) models accelerate hit-to-lead optimization by prioritizing compounds for synthesis from increasingly large chemical libraries. Classical machine learning architectures, including gradient-boosted ensembles, random forests, and feed-forward networks, have anchored ligand-based virtual screening for over a decade.^2,3^ Quantum machine learning (QML) has been proposed as a successor framework, motivated by the conjecture that encoding molecular descriptors into high-dimensional Hilbert spaces yields a more expressive inductive bias.^4–9^ Empirical evidence for this conjecture remains inconsistent across studies. Many early benchmarks used small descriptor sets that handicapped classical baselines or random train/test splits that allowed structurally similar compounds to appear in both sets, inflating apparent performance.^10,11^

The promise of QML is currently constrained by the noisy intermediate-scale quantum (NISQ) era, where hardware limitations force researchers to rely on simulators for benchmarking.^12^ Here, we benchmark two QML architectures—quantum support vector machine (QSVM) and variational quantum classifier (VQC)—against three classical baselines—gradient boosting classifier (GBC), random forest (RF), and neural network (NN)—across ten therapeutically diverse protein targets. We evaluate each model under Bemis–Murcko scaffold-stratified cross-validation^13^ and a multimetric scoring scheme, and we identify a target-level statistical signature that predicts when QML adds value.

Ten protein targets were selected for their established disease relevance, spanning neurodegenerative disease (BACE1), infectious disease (ACE2, Mtb DprE1, neuraminidase, reverse transcriptase), lysosomal storage disorder (NPC1), metabolic disease (insulin receptor, PCSK9), oncology (p53), and parasitic infection (Pf DHODH) (Table 1). Bioassay records from ChEMBL^14^ and BindingDB^15^ were standardized, deduplicated, filtered for assay reliability, and pruned of extreme measurements. The resulting data sets ranged from 45 (p53) to 18,286 (NPC1) compounds, consistent with known uneven distributions in target data.

**Table 1:** Target Information and Data Set Summary

Target	Associated Disease	Target Organism	N	pIC_50_ Thresholds
p53	cancer	*H. sapiens*	45	[5.50, 5.60, 5.80, 6.00]
ACE2	COVID-19	*H. sapiens*	149	[9.50, 9.58, 9.82]
Mtb DprE1	tuberculosis	*M. tuberculosis* (H37Rv)	172	[5.80, 6.00, 6.94, 7.00, 7.73, 8.00]
PCSK9	cardiovascular disease	*H. sapiens*	331	[5.51, 5.67, 5.90, 6.00]
Pf DHODH	malaria	*P. falciparum*	561	[5.50, 5.65, 5.90, 6.00, 7.00]
neuraminidase	influenza	Influenza A H1N1	619	[6.00, 6.07, 6.88, 7.00, 7.60, 8.00]
insulin receptor	type 2 diabetes	*H. sapiens*	2,359	[5.62, 6.00, 6.53, 7.00, 7.47, 8.00]
reverse transcriptase	HIV/AIDS	HIV-1	7,653	[5.40, 5.50, 5.65, 6.00]
BACE1	Alzheimer’s disease	*H. sapiens*	16,979	[5.39, 6.00, 6.43, 7.00, 7.49, 8.00]
NPC1	Niemann-Pick type C	*H. sapiens*	18,286	[5.45, 6.00, 7.00, 7.04, 8.00]

The five supervised classifiers (GBC, RF, NN, QSVM, VQC) were trained per target. Molecular representations comprised eight physicochemical descriptors and 2048-bit ECFP4 fingerprints generated with RDKit.^16,17^ A size-adaptive feature pipeline applied variance filtering, dimensionality reduction (top-variance selection, Tanimoto kernel PCA, or Jaccard-UMAP, depending on N), and 95%-variance PCA of physicochemical descriptors;^18,19^ for quantum models, a secondary PCA compressed features to 6–10 components to satisfy qubit-count constraints. Binary activity labels were assigned at multiple pIC_50_ thresholds per target. Full cross-validation partition rules and pipeline parameters are provided in the Experimental Procedures and Supporting Information.

Table 2 shows classical models had higher mean ROC-AUCs than quantum models. Additionally, the GBC achieved the highest mean Cohen’s κ, despite a lower ROC-AUC, suggesting that ranking and decision calibration capture different aspects of model utility. To establish whether observed rankings reflect genuine performance differences rather than sampling noise, Wilcoxon signed-rank tests were applied to all pairwise model comparisons across the 48 valid target-threshold pairs. The only statistically supported finding at α = 0.05 is that the NN outperforms GBC (W = 377, p = 0.048), RF (W = 349, p = 0.036), and QSVM (W = 388, p = 0.040). The NN versus VQC comparison approaches but does not cross the significance threshold (W = 400.5, p = 0.055). Critically, the aggregate comparison of best-quantum versus best-classical ROC-AUC across all target-threshold pairs is not statistically significant (W = 459, p = 0.189), meaning the observed tendency for classical models to rank higher cannot be distinguished from between-target sampling variation. All other pairwise comparisons—including GBC versus RF, GBC versus either quantum model, and RF versus either quantum model—are likewise nonsignificant. The global performance ranking in Table 2 should therefore be interpreted as an ordering of central tendencies under high between-target variance, not as a statistically confirmed hierarchy.

**Table 2:** Performance Summary by Model

Model	Mean ROC-AUC ± SD	Mean PR-AUC ± SD	Mean F1 ± SD	Mean Cohen’s κ ± SD	Mean Runtime (s)
NN	0.563 ± 0.095	0.494 ± 0.223	0.384 ± 0.347	0.037 ± 0.141	19.41 ± 21.89
RF	0.541 ± 0.100	0.485 ± 0.225	0.420 ± 0.279	0.033 ± 0.127	219.01 ± 205.67
GBC	0.532 ± 0.094	0.492 ± 0.215	0.432 ± 0.284	0.056 ± 0.145	1776.47 ± 1737.62
VQC	0.520 ± 0.093	0.473 ± 0.219	0.388 ± 0.274	0.022 ± 0.103	3681.73 ± 1888.39
QSVM	0.528 ± 0.084	0.486 ± 0.212	0.360 ± 0.348	0.018 ± 0.083	10294.22 ± 9437.47

Overall performance was modest across targets, with multiple models performing no better than random chance and mean ROC-AUC values over all models falling below 0.5 for ACE2 and Mtb DprE1 (Table 3). Performance near the discriminative floor illustrates the general difficulty of QSAR. The best-quantum/best-classical mean ROC-AUC gap (0.0345) is small relative to the across-model standard deviations (ranging from 0.093 to 0.100), indicating that target identity, not architecture, dominates performance variance (Table 3). These mean ROC-AUC values also reflect the rigorous nature of scaffold-based evaluation: random splitting consistently inflates performance estimates in QSAR by allowing structurally similar compounds to be included in both the training and test sets,^10,20–23^ and the results presented here are therefore expected to be more conservative than much of the published QSAR literature on these targets.

**Table 3:**
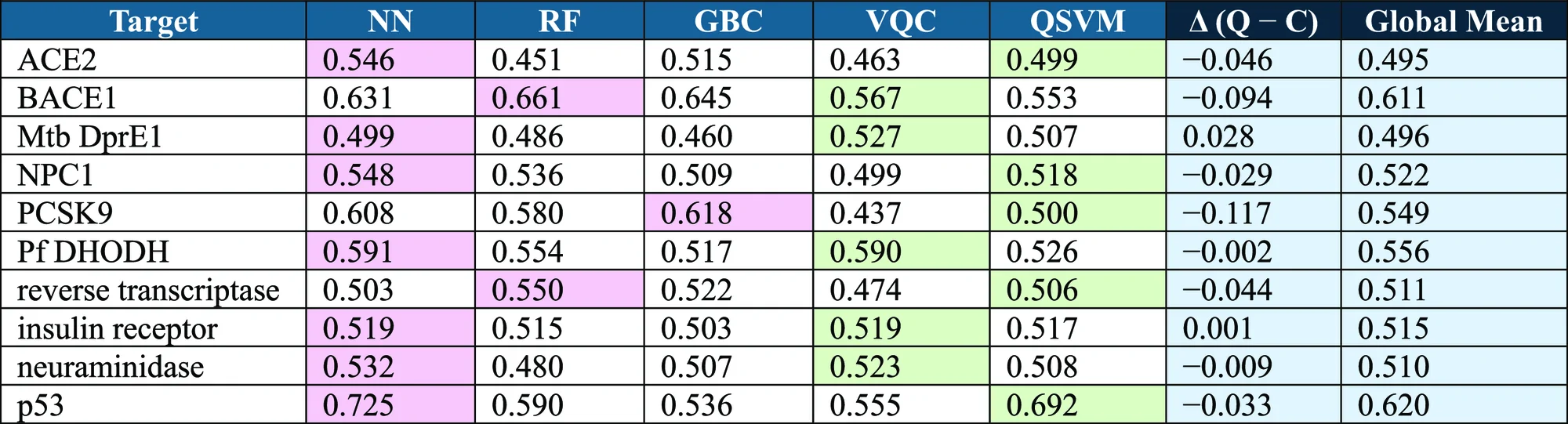
Mean ROC-AUC by Model and Target^a^

a **Note:** The pink shading indicates the highest mean ROC-AUC for each target across classical models. The green shading indicates the highest mean ROC-AUC for each target across quantum models. Δ (Q – C) is equal to the highest mean ROC-AUC across quantum models minus that across classical models for the given target. A perfect ROC-AUC was achieved by the QSVM on p53 at threshold 6.0; this is a degenerate evaluation artifact due to extreme test set imbalance and is excluded from all comparative analysis.

However, as seen in Figure 1, this poor performance is not correlated with data set size, with a negligible negative relationship between data size and performance. This suggests that chemical diversity and overall bioassay signal quality are the dominating limiting factors to performance in QSAR. The two best-performing targets, p53 and BACE1, are almost at completely opposite ends of the spectrum in terms of training set size, consistent with the notion that well-curated, target-focused data sets provide a more learnable structure than large and heterogeneous compound collections and that performance in a QSAR task is highly dependent on the target identity.

**Figure 1:**
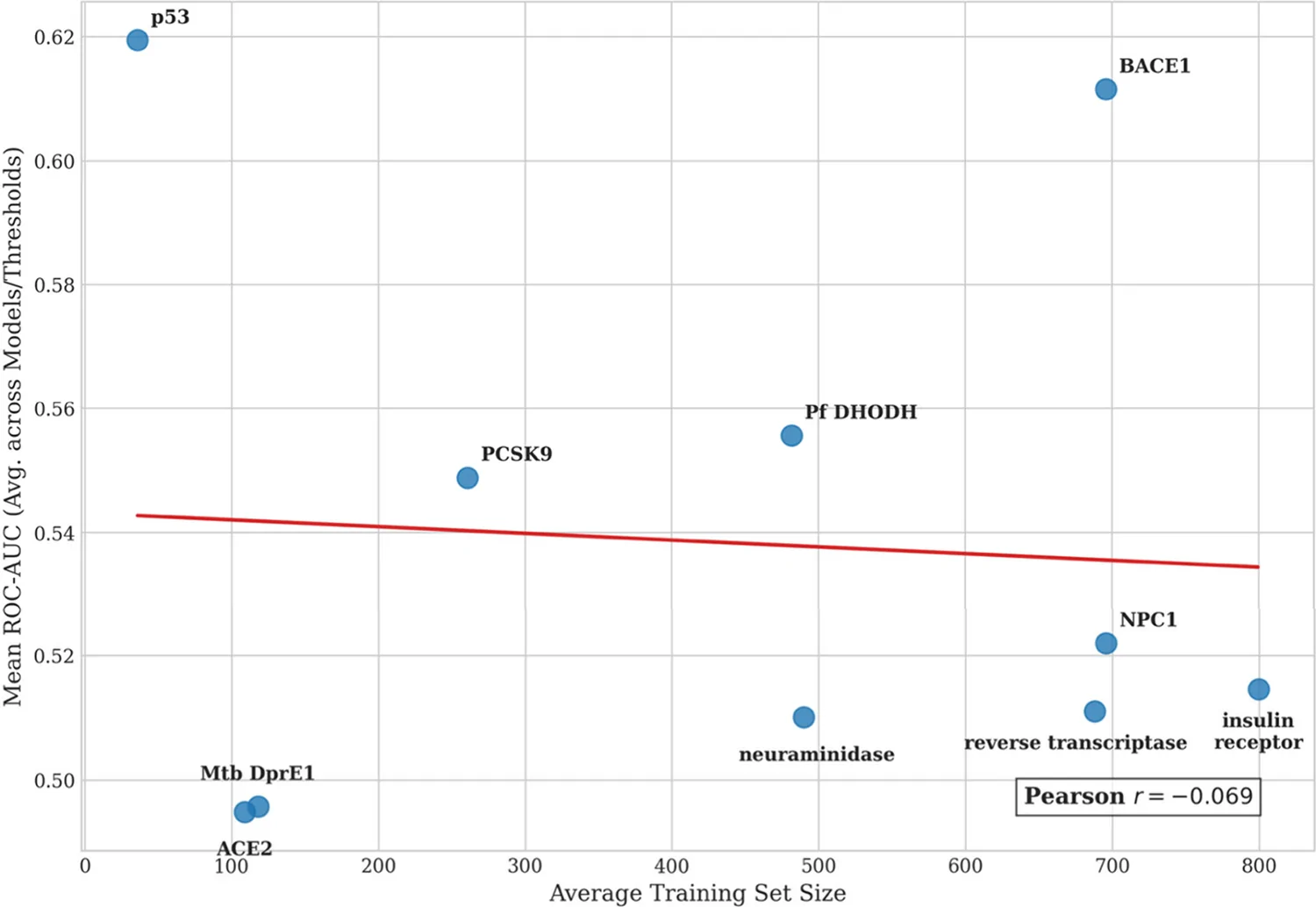
Correlation between training data set size and predictive performance. Target-level mean ROC-AUC (averaged across all models and applicable thresholds) is plotted against the average training set size for each of the ten protein targets. The red line represents a linear regression fit to the data. The Pearson correlation coefficient (r = −0.069) demonstrates that predictive success in this study is independent of raw data volume, suggesting that target-specific chemical diversity and bioassay signal quality are the primary drivers of model performance.

BACE1 exhibited the strongest classical model performance, with all three classical architectures achieving their maximum ROC-AUC at the pIC_50_ threshold of 7.49. This threshold is also pharmacologically meaningful for BACE1: inhibitor potency in the low-nanomolar range (pIC_50_ ≥ 7.49 corresponds to IC_50_ ≤ 32 nM) is generally required for *in vivo* efficacy, and this threshold effectively separates advanced lead compounds from weaker binders. The convergence of all three classical models to a similar, high ROC-AUC despite distinct architecture, combined with the ability of the classical models to detect the minority active class without triggering excessive false positives, as demonstrated in Figure 2, indicates that the classical models captured the true chemical signal of BACE1 inhibition rather than artificially inflating baseline accuracy by defaulting to the majority class. This convergent performance is best explained by the structural chemistry of potent BACE1 inhibitors. Nanomolar binders cluster on a small set of privileged transition-state-mimetic chemotypes that engage the catalytic aspartates Asp32 and Asp228,^24^ producing shared pharmacophoric fingerprint features across otherwise diverse lead series—a signal that classical tree ensembles and feed-forward networks are well suited to exploit. Scaffold-stratified splitting did not generate this signal; it is an evaluation safeguard against leakage, not a mechanism that improves generalization.

**Figure 2:**
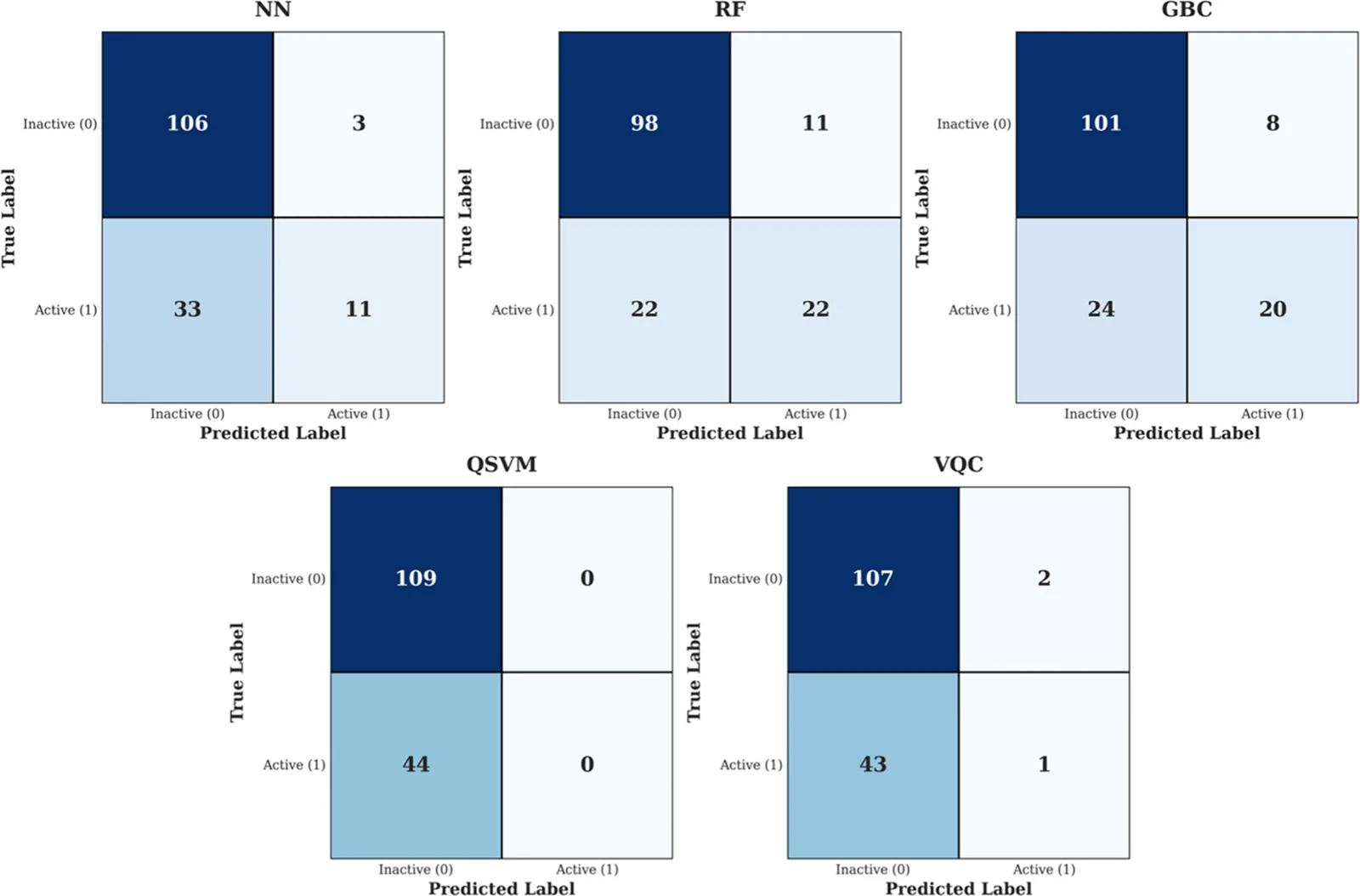
Confusion matrices for the classification of BACE1 inhibitors at the optimal pIC_50_ threshold of 7.49. The evaluated test set is imbalanced, comprising 109 inactive and 44 active compounds. Classical architectures (top row) successfully navigate this imbalance, detecting the minority active class while maintaining low false-positive rates. Conversely, quantum architectures (bottom row) exhibit nearly complete majority-class collapse, predicting the inactive class almost exclusively.

In contrast, quantum models underperformed on BACE1 at the 7.49 pIC_50_ threshold. The large discrepancy in ROC-AUC is best explained by the current limitations of quantum technology. Due to the quantum simulator’s boundaries, the quantum models were forced to operate on a 10-dimensional feature space (corresponding to 10 qubits) compared to the 75-dimensional input available to the classical models. This is an inherent constraint of the status quo in angle-encoded quantum classification. The behavioral impact of this severe information loss is starkly visible in the confusion matrices, showing underperformance is inextricably linked to the dimensionality bottleneck (Figure 2). Both quantum models suffered from nearly complete majority-class collapse. The severe dimensionality reduction stripped away the nuanced structural signal required to differentiate the rare active compounds from the inactive majority.

Quantum models outperformed all three classical models in 21 of 48 valid target-threshold combinations (44%), while classical models exceeded both quantum models by a margin greater than 0.10 ROC-AUC in 10 combinations (21%) (Figure 3). These figures establish that neither classical nor quantum approaches universally dominate: the comparative outcome is strongly target-specific, and the choice of computational tool should be informed by target characteristics rather than by a single cross-target ranking.

**Figure 3:**
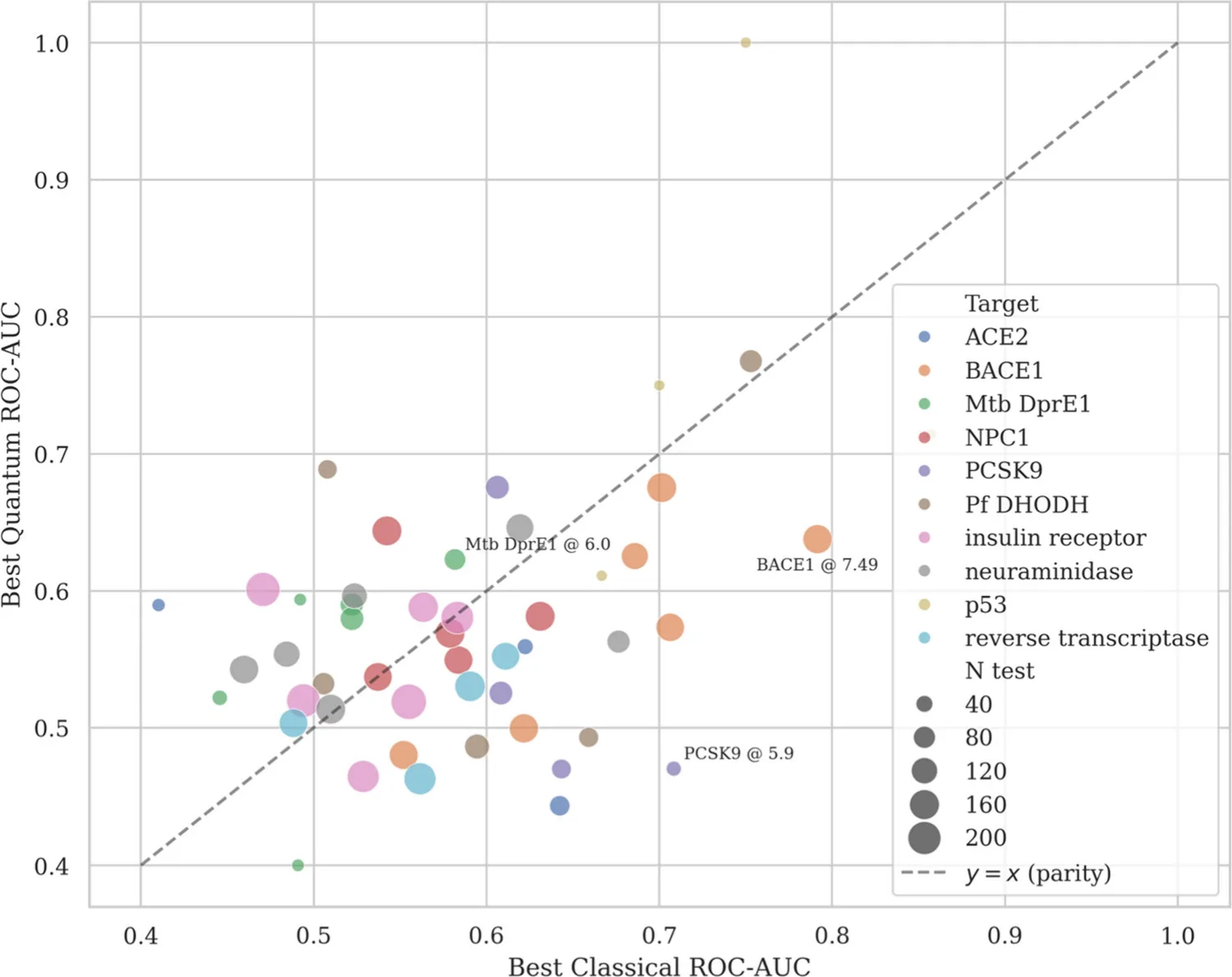
Comparative performance of optimal classical versus quantum architectures across all evaluated target-threshold tasks. Each marker represents a distinct data set configuration (target and pIC_50_ threshold). The coordinates correspond to the highest ROC-AUC achieved by any classical model on the *x*-axis and the highest ROC-AUC achieved by any quantum model on the *y*-axis for that specific task. The dashed parity line (y = x) delineates regions of classical advantage (below the line) and quantum advantage (above the line). Marker size is proportional to the test set size (N test), visually weighing the statistical confidence of the evaluation. Annotations highlight notable points of interest, as discussed earlier.

The clearest directional quantum advantage was observed for Mtb DprE1. Quantum models outperformed the best classical model at five of six evaluated thresholds, with VQC achieving the highest mean ROC-AUC for this target (Table 3). The consistent, albeit small, quantum advantage for Mtb DprE1 across thresholds, in the context of very small training data and a highly compressed feature space, suggests that the high-dimensional Hilbert-space kernel may capture structural relationships relevant to this target that are not accessible to the lower-dimensional classical representations, even with the classical models operating on larger feature spaces. This finding is consistent with observations from the drug-discovery QML literature that quantum classifiers can offer advantages over classical SVMs in low-data regimes, where kernel geometry matters more than representational depth. It also aligns with the p53 results, in which the critically limited data set size did not undermine the QSVM’s performance (Table 3). A chemical interpretation further supports this pattern: Mtb DprE1 inhibitors cluster into two structurally distinct families—nitroaromatic chemotypes (benzothiazinone, dinitrobenzamide) and non-nitro scaffolds (sulfonamide, benzimidazole, azaindole)—creating a binary-delineated chemical space well-suited to kernel-based classification in compressed feature representations.^25^ Per-target Wilcoxon testing across the Mtb DprE1 thresholds does not reach conventional significance (p = 0.313), so the quantum advantage should be considered a not yet statistically confirmed directional trend, pending validation on larger infectious disease compound sets.

Conversely, classical models were substantially superior for PCSK9 (mean quantum deficit: –0.117 ROC-AUC). For PCSK9, all five models show declining performance as the threshold increases, but the quantum models degrade faster: VQC achieves only 0.387 ROC-AUC at a threshold of 5.90, compared to NN’s 0.708 at the same threshold. The structural diversity of PCSK9 inhibitors (spanning small molecules, peptide mimetics, and allosteric binders) may require more feature dimensions than the 8–10-component quantum encoding provides, while classical models operating on 40–50-dimensional vectors retain sufficient representational capacity.

The observation that quantum advantage is target-specific rather than universal raises the question of which data set or target properties predict its occurrence. To address this, a correlation analysis was conducted between the per-target quantum advantage and four candidate predictors: mean training set size, active rate at the optimal threshold, number of evaluated thresholds (a proxy for assay coverage breadth), and the per-target Cohen’s κ gap (mean best-quantum κ – mean best-classical κ). Across the ten targets, training set size (Spearman ρ = +0.103, p = 0.770) and active rate (Spearman ρ = –0.067, p = 0.855) showed no meaningful correlation with quantum advantage. The number of evaluated thresholds (Spearman ρ = +0.530, p = 0.115) showed a moderate positive association, possibly reflecting that targets with richer assay coverage tend to have more structurally defined activity cliffs that quantum kernels can exploit. The strongest and only statistically significant predictor of per-target quantum advantage was the Cohen’s κ gap (Spearman ρ = +0.685, p = 0.029). Targets on which quantum models produce better-calibrated binary decisions relative to classical models also tend to be the targets on which quantum models achieve higher ROC-AUC.

This finding suggests that apparent quantum advantage is linked not only to ranking performance but also to whether the model produces useful binary decision boundaries under class imbalance. Practical guidance for target selection follows from this result: for a new target in an early discovery campaign, computing the κ gap on a small pilot data set using both classical and quantum classifiers may provide a low-cost screen for whether quantum methods will add value at scale. However, because this correlation is computed across only ten targets, the analysis is inherently underpowered, and these relationships should be interpreted as preliminary observations.

Computational profiling revealed a 530-fold mean runtime gap between the NN and the QSVM, driven by the inherent O­(N^2^) complexity of full statevector simulation for quantum kernel estimation and the AerSimulator’s statevector representation temporarily storing vectors exponential in qubit count (Figure 4). To prevent prohibitive runtimes, the VQC required a strict 500-compound training subset cap. While this introduced a data asymmetry for larger targets, comparative analysis confirmed this cap did not systematically suppress VQC performance or account for the architectural advantage of classical models on these data sets. Ultimately, scaling quantum kernel methods for real-world deployment will necessitate a transition from simulation to physical hardware execution or the development of specialized sampling strategies.

**Figure 4:**
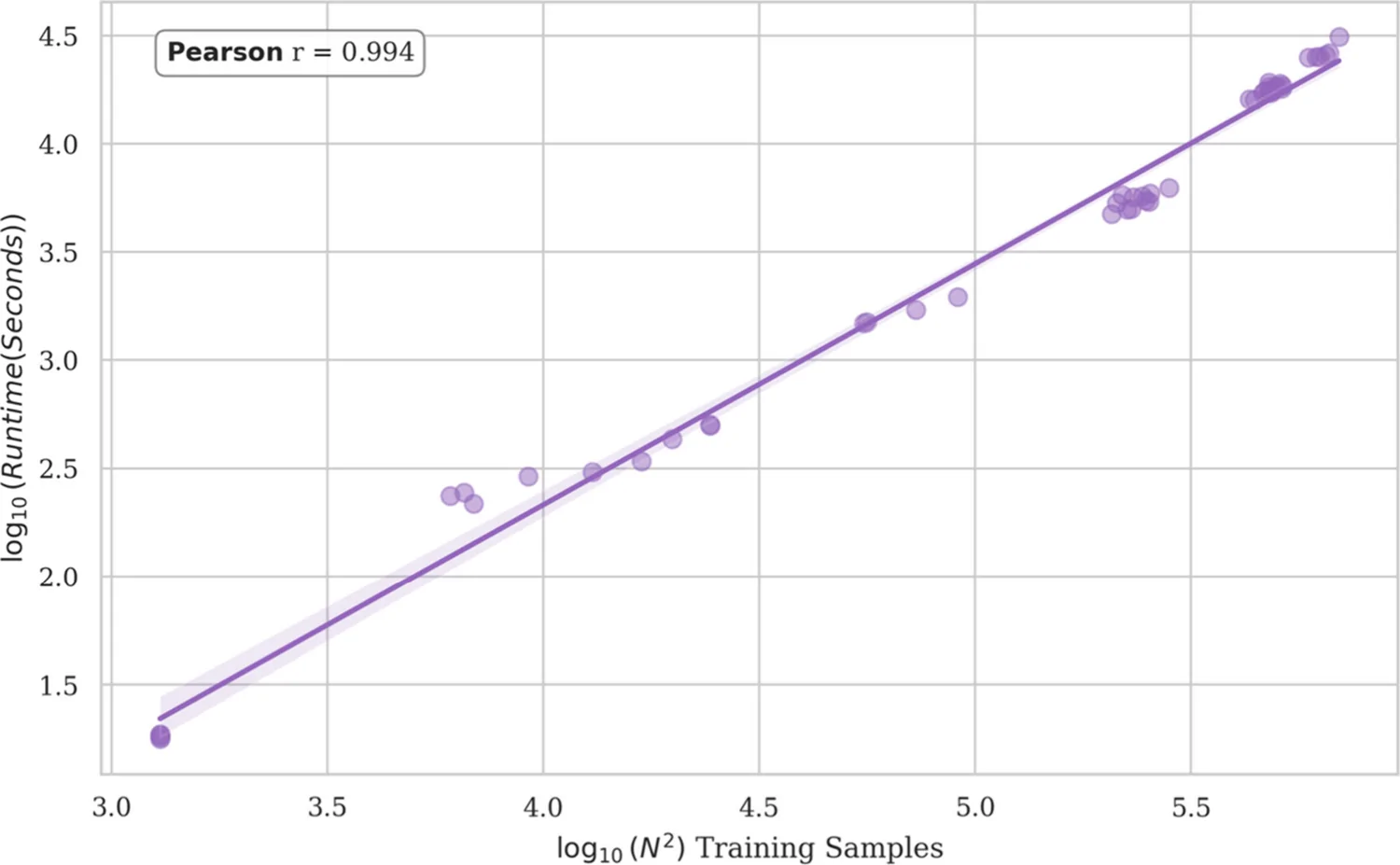
Empirical runtime scaling of the QSVM. The scatter plot demonstrates the computational time required to train the QSVM as a function of data set size. To evaluate the theoretical O­(N^2^) complexity associated with quantum kernel matrix evaluation, the base-10 logarithm of the execution time (in seconds) is plotted against the base-10 logarithm of the squared number of training samples log_10_(N^2^). A linear regression trendline is fitted to the data, with a strong Pearson r = 0.994, confirming O­(N^2^) scaling. All experiments were executed on the Georgia Tech ICE cluster on a single H100/H200 GPU node (64 CPU cores).

The findings from this benchmark establish a target-stratified framework for selecting computational tools in early stage drug discovery, indicating that neither classical nor quantum approaches universally dominate. For well-populated targets with structurally coherent compound series, such as BACE1, classical models are the recommended first-line choice. GBC achieved the strongest binary calibration and is preferred when reliable go/no-go classifications are required, with RF offering a faster alternative at modest calibration cost. The NN leads on ranking performance and runtime, making it better suited for automated, high-throughput workflows where speed and compound prioritization are favored over strict binary decisions.

For infectious disease targets characterized by sparse data, such as Mtb DprE1, quantum approaches demonstrated consistent advantages over classical methods, though overall predictive capacity on these targets remains close to random chance. This finding aligns with the theoretical capacity of quantum kernels to extract structural relationships within exponentially large Hilbert spaces, suggesting that quantum architectures warrant consideration for data-poor discovery contexts. However, this advantage is currently offset by substantial computational overhead. Because calculations were executed on a statevector simulator rather than physical quantum hardware, the simulated runtimes mask any potential quantum speedup. Scaling to the 1,000+ compound regime typical of mature screening campaigns will require physical-quantum execution or kernel-approximation methods. Furthermore, simulator outcomes represent an idealized upper bound because they do not account for real-world quantum errors, such as noise and decoherence.

For large, highly heterogeneous data sets (e.g., NPC1, RT), the uniform failure of all evaluated models to exceed near-random performance indicates that binary classification on the full compound space is not well-posed. The near-zero predictive capacity on NPC1, despite NPC1 containing over 18,000 total compounds (691 training compounds after scaffold-stratified partitioning), suggests a complex mixture of binding mechanisms that cannot be resolved through global models, pointing to the need for localized, chemotype-stratified modeling approaches.

These results are constrained by several methodological factors that inform future directions. Notably, the necessity of compressing molecular representations to satisfy quantum simulator constraints introduces an inherent asymmetry, as classical models operated on significantly richer feature spaces. This asymmetry indicates that any apparent quantum advantage or disadvantage is confounded by dimensionality differences. Future iterations utilizing amplitude encoding or quantum graph networks could facilitate fairer comparisons. Additionally, the reliance on binary classification, while useful for establishing potency thresholds, discards continuous pIC_50_ signals. Transitioning to regression formulations would eliminate threshold-dependent artifacts and provide a more robust measure of predictive capacity.

While the ML-guided prioritization of molecular property space effectively reduces the combinatorial burden of traditional screening, directly translating it into synthesis candidates remains computationally intensive. The generation of prioritized molecular descriptor profiles requires subsequent translation to specific chemical scaffolds, necessitating generative chemistry approaches or matched molecular pair analysis. Finally, while scaffold-stratified splitting mitigates the optimistic bias of random splits, prospective validation on synthesized compounds will be required to rigorously confirm generalization performance. As quantum hardware advances toward error-corrected systems and software infrastructure matures, periodic reassessment of these computational paradigms will be essential to accurately gauge their role in computational drug discovery.

## Experimental Procedures

Models were evaluated on ROC-AUC as the primary ranking metric; Cohen’s κ and F1 measured binary decision behavior and detected majority-class collapse under class imbalance, and runtime was recorded in seconds. PR-AUC was reported to provide an imbalance-sensitive complement to ROC-AUC.

Bioassay records from BindingDB and ChEMBL were standardized, deduplicated, and filtered for assay reliability to yield SMILES–IC_50_ pairs, which were converted to pIC_50_. RDKit generated 2048-bit ECFP4 fingerprints (diameter = 4) and eight physicochemical descriptors per compound: molecular weight, LogP, topological polar surface area, Labute approximate surface area, hydrogen-bond donors, hydrogen-bond acceptors, rotatable bonds, and ring count.

Features were processed through a size-adaptive three-stage pipeline: (1) variance filtering of fingerprint bits with a data set-size-dependent threshold (Table S2); (2) dimensionality reduction by top-variance selection (*N* ≤ 50), Tanimoto kernel PCA (50 < *N* ≤ 500), or Jaccard-metric UMAP (*N* > 500); and (3) 95%-variance PCA of physicochemical descriptors. For quantum models, a secondary PCA compressed features to 6, 8, or 10 components for *N* ≤ 50, 50 < *N* ≤ 5,000, and *N* > 5,000, respectively, to satisfy qubit-count constraints.

Binary activity labels were generated by combining pharmacological pIC_50_ anchors (6.00, 7.00, 8.00) with data set quartiles; thresholds were retained only when both classes contained at least five compounds, yielding 49 target–threshold combinations and 245 model-level results. All models within a given target-threshold combination were evaluated on an identical data partition. Partitions followed a size-adaptive scheme: 5-fold stratified CV (80/20) for *N* ≤ 50, 3-fold Bemis–Murcko scaffold-stratified CV (80/20) for 50 < *N* ≤ 5,000, and a fixed 70/15/15 scaffold-stratified split for *N* > 5,000. Per-threshold N_train_ and N_test_ values are listed in Table S1.

Three classical models (GBC, RF, NN) were implemented in scikit-learn and two quantum models (QSVM, VQC) were implemented in Qiskit, with quantum circuits executed on the AerSimulator (statevector method) with GPU acceleration.^26–31^ Specific architectural configurations and computational constraints were tailored to each model’s underlying mechanics and the scale of the data set. Among the classical architectures, the NN progressed from shallow, single-layer topologies for small data sets to broader, two-layer configurations for larger target evaluations, incorporating L2 regularization penalties and a maximum of 1,000 training iterations to mitigate overfitting. For the tree-based ensembles, the RF inherently addressed data set class imbalance via balanced class weighting, while the GBC utilized stochastic subsampling and minimum impurity decrease thresholds. Quantum models required strict data set capping to accommodate the intense computational overhead of statevector simulation: the QSVM was restricted to 1,000 training samples due to the O­(N^2^) scaling of its FidelityQuantumKernel matrix evaluation (utilizing a fixed 2-repetition ZZFeatureMap), while the VQC was capped at 500 samples. The VQC dynamically optimized its architecture by selecting between various data-encoding feature maps and RealAmplitudes variational ansatzes using the simultaneous perturbation stochastic approximation (SPSA) optimizer over 100 iterations. Notably, fresh quantum circuit objects were initialized for every VQC training iteration to definitively prevent warm-start bias contamination during optimization. Classical models were tuned via stratified double-loop nested cross-validation while quantum models, owing to the exponential runtime cost of statevector simulation, used a fixed hold-out validation partition. Search spaces and optimized configurations are reported in Tables S3 and S4, respectively. Code is available at https://github.com/shiv2158/potency_prediction_model_evaluation.

No unexpected or unusually high safety hazards were encountered.

## Supplementary Material



## Notes

Additional experimental details, including train and test sample sizes by pIC_50_ threshold value (Table S1), variance filtering thresholds for ECFP4 fingerprint bits based on data set size (Table S2), hyperparameter search spaces for each model based on data set size (Table S3), and optimized hyperparameter configurations for each model-target combination dependent on the potency thresholds with the highest overall ROC-AUC (Table S4) (PDF)

## Abbreviations

ACE2: angiotensin-converting enzyme 2
AUC: area under the curve
BACE1: beta-site amyloid precursor protein cleaving enzyme 1
CPU: computer processing unit
ECFP4: extended-connectivity fingerprint with diameter 4
GB: gigabytes
GBC: gradient boosting classifier
GPU: graphics processing unit
HBA: hydrogen bond acceptors
HBD: hydrogen bond donors
MB: megabytes
IC_50_: half-maximal inhibitory concentration
ML: machine learning
Mtb DprE1: *M. tuberculosis* decaprenylphosphoryl-β-D-ribose-2’-epimerase 1
MW: molecular weight
NISQ: noisy intermediate-scale quantum
NN: neural network
NPC1: Niemann-Pick C1
PACE: Partnership for an Advanced Computing Environment
PCA: principal component analysis
PCSK9: proprotein convertase subtilisin/kexin type 9
Pf DHODH: *P. falciparum* dihydroorotate dehydrogenase
pIC_50_: negated base-10 logarithm of half-maximal inhibitory concentration
PR: precision-recall curve
QC: quantum computing
QML: quantum machine learning
QSAR: quantitative structure–activity relationship
QSVM: quantum support vector machine
RF: random forest
ROC: receiver operating characteristic curve
RT: reverse transcriptase
SD: standard deviation
SMILES: simplified molecular input line entry system
SPSA: simultaneous perturbation stochastic approximation
UMAP: uniform manifold approximation and projection
VQC: variational quantum classifier
