# Supplementary material for: Benchmarking Classical and Quantum Machine Learning for Potency Prediction across Ten Therapeutically Relevant Targets
Source: ACS Med Chem Lett. 2026 Jul 7;17(8):1736–43. doi: 10.1021/acsmedchemlett.5c00724 (PMC13488102; doi:10.1021/acsmedchemlett.5c00724)
Supplement: Supplementary file 1 [file ml5c00724_si_001.pdf]

Benchmarking Classical and Quantum Machine Learning for Potency Prediction across Ten Therapeutically Relevant Targets

Shiv Garg*[1] and Sara Garg[2]

[1]Georgia Institute of Technology, Atlanta, Georgia 30332, United States; Email: sgarg349@gatech.edu

[2]Emory University, Atlanta, Georgia 30322, United States

Supporting Information

Table S1. Per-Threshold Train and Test Sample Sizes

Target	pIC_{50} Threshold	N_{train}	N_{test}
p53	5.50	36	9
p53	5.60	36	9
p53	5.80	36	9
p53	6.00	36	9
ACE2	9.50	83	66
ACE2	9.58	114	35
ACE2	9.82	130	19
Mtb DprE1	5.80	141	31
Mtb DprE1	6.00	96	76
Mtb DprE1	6.94	156	16
Mtb DprE1	7.00	81	91
Mtb DprE1	7.73	156	16
Mtb DprE1	8.00	78	94
PCSK9	5.51	237	94
PCSK9	5.67	270	61
PCSK9	5.90	302	29
PCSK9	6.00	235	96
Pf DHODH	5.50	479	82
Pf DHODH	5.65	503	58
Pf DHODH	5.90	455	106
Pf DHODH	6.00	498	63
Pf DHODH	7.00	474	87
neuraminidase	6.00	504	115
neuraminidase	6.07	530	89
neuraminidase	6.88	494	125
neuraminidase	7.00	468	151
neuraminidase	7.60	461	158
neuraminidase	8.00	482	137
insulin receptor	5.62	813	187
insulin receptor	6.00	769	231
insulin receptor	6.53	834	166

insulin receptor	7.00	792	208
insulin receptor	7.47	805	195
insulin receptor	8.00	785	215
reverse transcriptase	5.40	681	167
reverse transcriptase	5.50	717	143
reverse transcriptase	5.65	657	186
reverse transcriptase	6.00	697	146
BACE1	5.39	666	149
BACE1	6.00	693	152
BACE1	6.43	716	128
BACE1	7.00	703	144
BACE1	7.49	713	153
BACE1	8.00	685	160
NPC1	5.45	691	149
NPC1	6.00	707	157
NPC1	7.00	693	159
NPC1	7.04	696	145
NPC1	8.00	692	159

Note: Test sizes vary across thresholds even within the same target because Bemis–Murcko scaffold groups are unequal in size, and binary label rebalancing at each pIC_{50} threshold redistributes compounds across folds.

Table S2. Variance Filtering Thresholds for ECFP4 Fingerprint Bits

Dataset Size (N)	Bernoulli Variance Threshold (p × (1-p))
N ≤ 50	0.10 × 0.90
50 < N ≤ 200	0.07 × 0.93
200 < N ≤ 500	0.06 × 0.94
500 < N ≤ 5,000	0.05 × 0.95
N > 5,000	0.03 × 0.97

Note: Bits with Bernoulli variance below the listed product (the variance of a binary feature with active fraction p) were removed. Stricter thresholds at small N preserve discriminative bits that would otherwise be lost to sampling noise; looser thresholds at large N control the curse of dimensionality.

Table S3. Summary of Hyperparameter Search Spaces by Dataset Size

Model	N ≤ 50	50 < N ≤ 500	500 < N ≤ 1000	N > 1000
NN	hidden layer sizes: {(16,), (32,)} alpha: {0.001, 0.01}	hidden layer sizes: {(32,), (64,), (32, 16)} alpha: {0.0001, 0.001}	hidden layer sizes: {(64,), (128,), (64, 32)} alpha: {0.0001, 0.001}	hidden layer sizes: {(128,), (256,), (128, 64), (256, 128)} alpha: {0.00001, 0.0001, 0.001}
RF	n estimators: {100, 200} max depth: {2, 3} min samples leaf: {3, 5}	n estimators: {100, 200, 500} max depth: {3, 5, 10} min samples leaf:	n estimators: {100, 200, 500} max depth: {3, 5, 10, None} min samples leaf: {1, 2,	n estimators: {200, 500, 1000} max depth: {5, 10, 20, None} min samples leaf: {1, 2,

	max features: {sqrt}	{1, 2, 4}<br>max features: {sqrt, log2}	4}<br>max features: {sqrt, log2}	4}<br>max features: {sqrt, log2}
GBC	n estimators: {100, 200}<br>learning rate: {0.05, 0.1}<br>max depth: {2, 3}	n estimators: {100, 200}<br>learning rate: {0.01, 0.05, 0.1}<br>max depth: {2, 3, 4}<br>subsample: {0.8, 1.0}	n estimators: {100, 200, 500}<br>learning rate: {0.01, 0.05, 0.1}<br>max depth: {2, 3, 4, 5}<br>subsample: {0.6, 0.8, 1.0}<br>min impurity decrease: {0, 0.001}	n estimators: {200, 500, 1000}<br>learning rate: {0.01, 0.05, 0.1}<br>max depth: {3, 4, 5}<br>subsample: {0.6, 0.8, 1.0}<br>min impurity decrease: {0, 0.001, 0.01}
VQC	architecture: {zf ra1 linear, zf ra2 linear, zzf ra1 full, zzf ra2 linear}			
QSVM	C: {0.1, 1.0, 10.0, 100.0}			

Note: The classical models used a stratified nested cross-validation protocol to search hyperparameters, with an outer scaffold-aware split for evaluation and an inner stratified K-fold split for tuning to address class imbalance. The quantum models instead used a hold-out set search protocol for computational efficiency, employing a dedicated, separate validation partition for hyperparameter tuning.

Table S4. Optimized Hyperparameter Configurations for each Model-Target Combination at the Highest Performing Potency Thresholds

Target	NN	RF	GBC	VQC	QSVM
p53	threshold: 5.80<br>hidden layer sizes: [32]<br>alpha: 0.001	threshold: 5.80<br>n estimators: 200<br>max depth: 2<br>min samples leaf: 3<br>max features: sqrt	threshold: 6.00<br>n estimators: 100<br>learning rate: 0.1<br>max depth: 3	threshold: 5.60<br>architecture: zzf ra1 full	threshold: 5.60<br>C: 10.0
ACE2	threshold: 9.50<br>hidden layer sizes: [64]<br>alpha: 0.0001	threshold: 9.58<br>n estimators: 200<br>max depth: 5<br>min samples leaf: 2<br>max features: sqrt	threshold: 9.58<br>n estimators: 200<br>learning rate: 0.1<br>max depth: 3<br>subsample: 0.8	threshold: 9.58<br>architecture: zzf ra1 full	threshold: 9.82<br>C: 0.1
Mtb DprE1	threshold: 6.00<br>hidden layer sizes: [32, 16]<br>alpha: 0.0001	threshold: 6.00<br>n estimators: 100<br>max depth: 5<br>min samples leaf: 2<br>max features: sqrt	threshold: 6.00<br>n estimators: 100<br>learning rate: 0.1<br>max depth: 4<br>subsample: 0.8	threshold: 6.00<br>architecture: zf ra1 linear	threshold: 6.94<br>C: 100.0
PCSK9	threshold: 5.90<br>hidden layer sizes: [32]<br>alpha: 0.0001	threshold: 5.90<br>n estimators: 100<br>max depth: 10<br>min samples leaf: 1<br>max features: sqrt	threshold: 5.67<br>n estimators: 200<br>learning rate: 0.05<br>max depth: 2<br>subsample: 0.8	threshold: 5.51<br>architecture: zzf ra2 linear	threshold: 6.00<br>C: 0.1
Pf DHODH	threshold: 7.00<br>hidden layer sizes: [64, 32]<br>alpha: 0.0001	threshold: 7.00<br>n estimators: 100<br>max depth: 10<br>min samples leaf: 4<br>max features: sqrt	threshold: 7.00<br>n estimators: 200<br>learning rate: 0.1<br>max depth: 3<br>subsample: 0.6<br>min impurity decrease: 0.001	threshold: 7.00<br>architecture: zzf ra1 full	threshold: 5.65<br>C: 100.0
neuraminidase	threshold: 6.07<br>hidden layer	threshold: 8.00<br>n estimators: 100<br>max depth: None	threshold: 8.00<br>n estimators: 100<br>learning rate: 0.05	threshold: 8.00<br>architecture: zf ra1 linear	threshold: 6.07<br>C: 100.0

	sizes: [64, 32]<br>alpha: 0.001	min samples leaf: 1<br>max features: sqrt	max depth: 4<br>subsample: 1.0<br>min impurity decrease: 0.001		
insulin receptor	threshold: 6.53<br>hidden layer sizes: [128, 64]<br>alpha: 0.00001	threshold: 7.47<br>n estimators: 500<br>max depth: 5<br>min samples leaf: 1<br>max features: log2	threshold: 6.00<br>n estimators: 200<br>learning rate: 0.05<br>max depth: 3<br>subsample: 0.6<br>min impurity decrease: 0.0	threshold: 8.00<br>architecture: zzf ra2 linear	threshold: 7.47<br>C: 0.1
reverse transcriptase	threshold: 5.50<br>hidden layer sizes: [256, 128]<br>alpha: 0.00001	threshold: 5.50<br>n estimators: 200<br>max depth: 10<br>min samples leaf: 2<br>max features: sqrt	threshold: 5.50<br>n estimators: 500<br>learning rate: 0.01<br>max depth: 3<br>subsample: 0.8<br>min impurity decrease: 0.01	threshold: 5.50<br>architecture: zf ra1 linear	threshold: 5.50<br>C: 1.0
BACE1	threshold: 7.49<br>hidden layer sizes: [256]<br>alpha: 0.001	threshold: 8.00<br>n estimators: 500<br>max depth: None<br>min samples leaf: 4<br>max features: log2	threshold: 7.49<br>n estimators: 500<br>learning rate: 0.01<br>max depth: 3<br>subsample: 0.6<br>min impurity decrease: 0.001	threshold: 7.49<br>architecture: zf ra2 linear	threshold: 8.00<br>C: 1.0
NPC1	threshold: 7.00<br>hidden layer sizes: [256, 128]<br>alpha: 0.00001	threshold: 5.45<br>n estimators: 500<br>max depth: 5<br>min samples leaf: 1<br>max features: sqrt	threshold: 6.00<br>n estimators: 1000<br>learning rate: 0.1<br>max depth: 4<br>subsample: 0.8<br>min impurity decrease: 0.0	threshold: 7.00<br>architecture: zzf ra2 linear	threshold: 8.00<br>C: 0.1

Note: Optimal configurations were selected based on the highest achieved ROC-AUC across all evaluated pIC_{50} thresholds for each model-target combination. For VQC, the architecture combines a feature map (the quantum embedding circuit used for data encoding) with a variational ansatz.
